# Homebound by COVID19: the benefits and consequences of non-pharmaceutical intervention strategies

**DOI:** 10.1186/s12889-021-10725-9

**Published:** 2021-04-06

**Authors:** Buse Eylul Oruc, Arden Baxter, Pinar Keskinocak, John Asplund, Nicoleta Serban

**Affiliations:** 1 H. Milton Stewart School of Industrial and Systems Engineering, Georgia Institute of Technology, North Ave NW, Atlanta, GA 30332 USA; 2 Department of Environmental Health, Rollins School of Public Health, Emory University, Atlanta, GA USA; 3 Metron, Inc., 1818 Library Street #600, Reston, VA USA

**Keywords:** Agent-based disease modeling, Non-pharmaceutical intervention strategies, COVID19

## Abstract

**Background:**

Recent research has been conducted by various countries and regions on the impact of non-pharmaceutical interventions (NPIs) on reducing the spread of COVID19. This study evaluates the tradeoffs between potential benefits (e.g., reduction in infection spread and deaths) of NPIs for COVID19 and being homebound (i.e., refraining from interactions outside of the household).

**Methods:**

An agent-based simulation model, which captures the natural history of the disease at the individual level, and the infection spread via a contact network assuming heterogeneous population mixing in households, peer groups (workplaces, schools), and communities, is adapted to project the disease spread and estimate the number of homebound people and person-days under multiple scenarios, including combinations of shelter-in-place, voluntary quarantine, and school closure in Georgia from March 1 to September 1, 2020.

**Results:**

Compared to no intervention, under voluntary quarantine, voluntary quarantine with school closure, and shelter-in-place with school closure scenarios 4.5, 23.1, and 200+ homebound adult-days were required to prevent one infection, with the maximum number of adults homebound on a given day in the range of 119 K–248 K, 465 K–499 K, 5388 K-5389 K, respectively. Compared to no intervention, school closure only reduced the percentage of the population infected by less than 16% while more than doubling the peak number of adults homebound.

**Conclusions:**

Voluntary quarantine combined with school closure significantly reduced the number of infections and deaths with a considerably smaller number of homebound person-days compared to shelter-in-place.

**Supplementary Information:**

The online version contains supplementary material available at 10.1186/s12889-021-10725-9.

## Background

Recent research and experiences from various communities around the world highlighted the potential benefits of non-pharmaceutical interventions (NPIs) for slowing down the spread of COVID19 and reducing the severe health outcomes [1–3]. NPIs include school closure, reducing public gatherings, social distancing, restricting travel, and voluntary quarantine (entire household staying at home if someone in the household has symptoms) [4–7] and more stringent interventions such as shelter-in-place [8, 9].

People may become “homebound” (i.e., stay home and refrain from interactions in the community/workplace) due to complying with some of the NPIs (even if they do not experience symptoms), showing symptoms, or providing childcare. Hence, despite their benefits, there are also unintended consequences of NPIs, including the impact on the economy, unemployment, household spending, mobility, energy usage, etc. [10–13] and the social impact on caring for the elderly, education of the young, family support, domestic violence, and personal health and wellbeing [14–23].

Some NPIs, such as shelter-in-place, apply to large populations for an extended duration, whereas others, such as voluntary quarantine, impact targeted populations for a limited time. It is important to understand the tradeoffs between the public health benefits and other consequences of NPIs, particularly, as measured by homebound person-days or the size of the homebound population over time. There is sparse research on assessing which interventions have a higher overall impact in reducing societal interactions versus the ability to reduce infection spread and adverse outcomes [8, 9, 24, 25].

This study evaluates the trade-offs between the public health impact measures (e.g., the number of cases, hospitalizations and deaths [26]) and intervention metrics, including number of homebound people and person-days under various NPI scenarios, including variations of shelter-in-place, voluntary quarantine, and school closure. The intervention metrics aim to capture how much an intervention reduces societal activity and interaction, much needed to maintain economic and social life. Such evaluations can assist local and national decision makers in choosing different combinations of targeted interventions over time to reduce infection spread while considering the societal and economic impact.

In this paper, we use the homebound person-days as a proxy for the decrease in the economic and social activity due to COVID19, and test the following hypotheses using a simulation model.

- **Hypothesis 1:** Voluntary quarantine (with school closure) leads to a significant reduction in the number of COVID19 infections.
- **Hypothesis 2:** Shelter-in-place significantly increases the number of homebound person-days, with limited impact on the number of COVID19 infections.

## Methods

### Intervention definitions

**Fig. 1:**
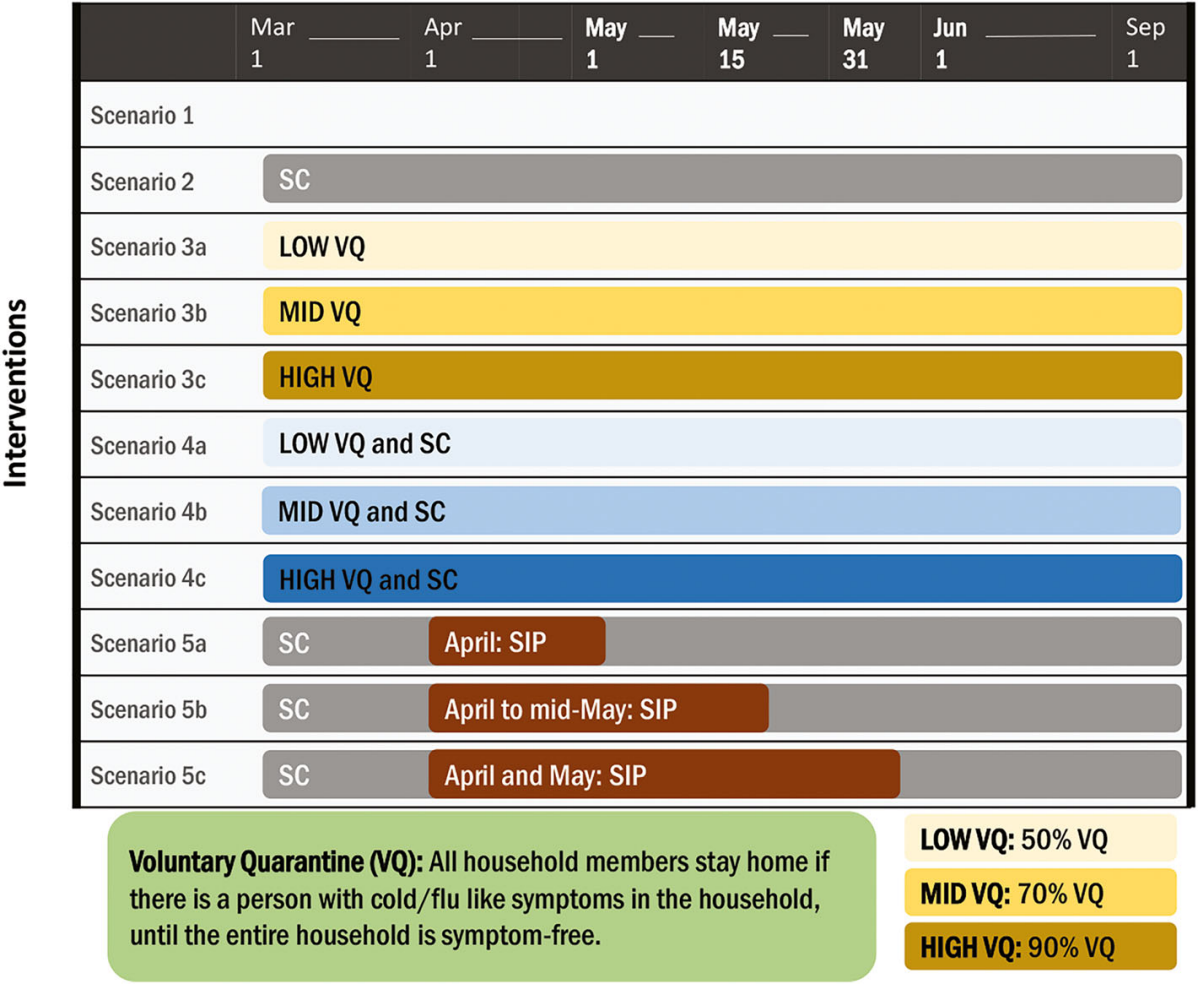
Scenarios. Description of the intervention scenarios considered in this study

The following NPIs, with varying combinations and compliance levels in different scenarios (Fig. 1), are analyzed in this study and compared to the baseline of no intervention (NI):

1. *School Closure (SC)* – No peer-group interactions among children or youth (i.e., no K-12 school interactions).
2. *Voluntary Quarantine (VQ)* –Household members stay home if any member of the household is symptomatic, until the entire household is symptom-free.
3. *Shelter-in-Place (SIP)* – Household members stay home.

### Modeling case projection and estimating intervention impact

An agent-based simulation model with heterogeneous population mixing was utilized and adapted, which has been previously applied to project the number of COVID19 infections and severe outcomes under various social distancing strategies [26]. The simulation model was implemented using C++. The underlying disease progression model was a variant of a Susceptible-Exposed-Infected-Recovered (SEIR) model. The model tracks the disease status of an individual while the disease spreads through the contact networks consisting of households, peer groups (workplaces, schools), and communities. Each individual was assumed to be either susceptible (S), exposed (E), transitioning (IT), asymptomatic (IA), symptomatic (IS), hospitalized (H), recovered (R), or dead (D) at any given time. Further, population mixing was assumed to be in (i) households (night), (ii) peer groups (day), and (iii) communities (day and night). (A more detailed model description and model parameters can be found in [26]). The study period is March 1, 2020- September 1, 2020. All results presented in this study are the averages of 30 replications (for each scenario) of the agent-based simulation model runs.

The population in the simulation includes children (ages 0–9), youth (ages 10–19), adults (ages 20–64), and elderly (ages 65+). The simulation monitors the health status (e.g., symptomatic, hospitalized, dead) as well as the homebound status of each household member (for further details see Supplementary Section A, Additional file 1 and Supplementary Table 1, Additional file 1).

- Homebound: For adults and elderly, this status is defined as staying home due to voluntary quarantine, symptoms, shelter-in-place, or *at home childcare*, i.e., providing supervision to a child who is home due to their status (e.g., due to symptoms or school closure). For example, if a child is at home in need of supervision, the status of an adult or elderly member in the household is updated to indicate that they provide supervision, labeled as *at home childcare*. For children and youth, homebound is defined as staying home due to voluntary quarantine, symptoms, or school closure.
- Inactive: For adults and elderly, a status of inactive refers to being inactive from society due to being homebound, hospitalized, or providing hospital care, i.e., caring for a child or youth who became hospitalized. A status of inactive for children and youth is defined as being inactive from society due to being homebound or hospitalized.

### Infection spread outcome measures and intervention metrics

The infection spread outcome measures reported for the study period include:

- Cumulative deaths: Number of people who died due to COVID19.
- Cumulative infections: Number of people infected (including asymptomatic infections).
- Peak day: The day when the number of new infections was highest.
- Peak infection: The highest number (or percentage) of the population infected on a given day.

A statistical summary of infection spread outcome measures under baseline and intervention scenarios is provided in Supplementary Table 2, Additional file 1.

The infection spread measures are contrasted with the following intervention metrics, which are reported for the study period:

- Homebound or inactive subpopulation: Number of people in a subpopulation (adults/elderly or children/youth) with homebound or inactive status, respectively, on a given day.
- Percentage of days adults homebound or inactive: Average percentage of days an adult has homebound or inactive status, respectively.
- Homebound days: Average number of days a (sub) population has homebound status.
- Homebound or inactive peak day: The day when the number of a (sub) population has homebound or inactive status, respectively, is highest.
- Homebound or inactive peak: The highest number (or percentage) of a (sub) population homebound or inactive, respectively, on a given day.
- Adults absent from work: The number of adults who are absent from work due to an inactive status (further details are provided in Supplementary Section B, Additional file 1).
- Homebound days to prevent an infection: Additional adult homebound days needed to prevent an infection (in Scenario X, relative to Scenario 1), calculated as follows:
  $$ \frac{Adult\ Homebound\ Days\ in\ Scenario\ X- Adult\ Homebound\ Days\ in\ Scenario\ 1\ }{Cumulative\ Infections\ in\ Scenario\ 1- Cumulative\ Infections\ in\ Scenario\ X} $$
- Homebound days to prevent a death: Additional adult homebound days needed to prevent a death (in Scenario X, relative to Scenario 1), calculated as follows:
  $$ \frac{Adult\ Homebound\ Days\ in\ Scenario\ X- Adult\ Homebound\ Days\ in\ Scenario\ 1\ }{Cumulative\ Deaths\ in\ Scenario\ 1- Cumulative\ Deaths\ in\ Scenario\ X} $$

An ethics approval for this study has been deemed not applicable as all data used is publicly available from census information.

## Results

**Fig. 2:**
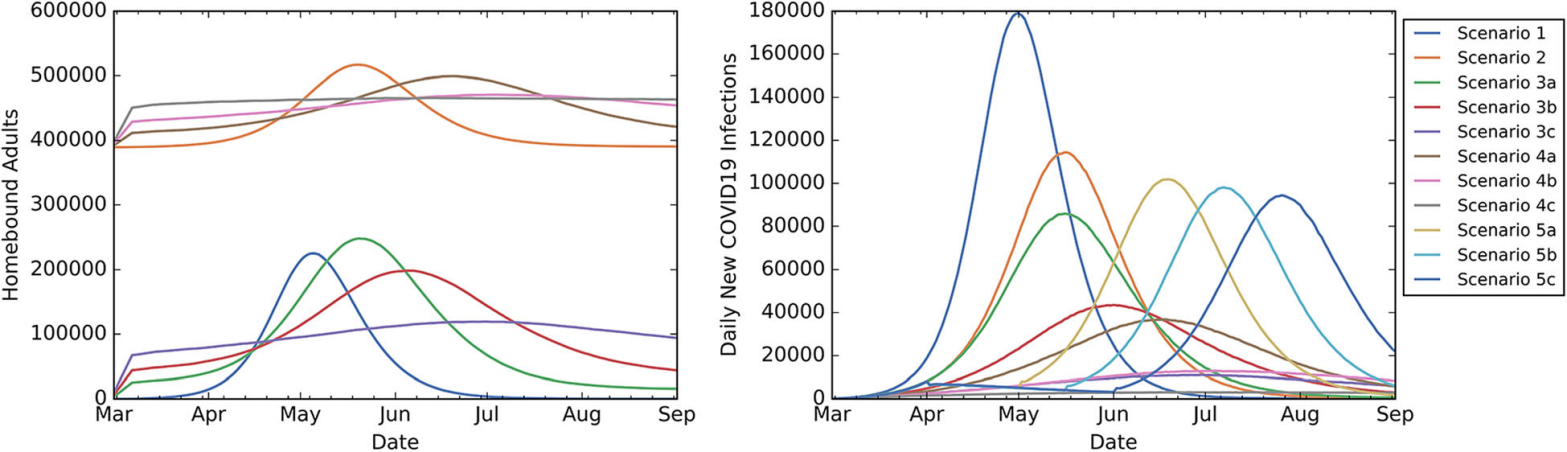
Homebound adults and daily new infections. Homebound adults and daily new infections over time. Scenarios 2, 4a, 4b, 4c include school closure

Figure 2 presents the daily new infections and the homebound adults over time across all scenarios. Under Scenarios 1, 3a, 3b, 3c (non-school closure scenarios), the homebound peak for adults decreased from 248,421 under Scenario 3a to 119,461 under Scenario 3c, and the peak under Scenario 1 was 225,315. Under Scenarios 2, 4a, 4b, 4c, 5a, 5b, 5c (school closure scenarios), the homebound peak for adults was highest under Scenarios 5a, 5b, 5c, due to shelter-in-place, ranging from 5,388,074 to 5,389,220, followed by homebound peak of 516,870 under Scenario 2 (see Supplementary Table 4, Additional file 1 to compare homebound peak percentages for adults). Adults absent from work followed a similar pattern as homebound adults across all scenarios (Supplementary Figure 1, Additional file 1).

Higher compliance with voluntary quarantine reduced homebound peak for adults to 499,188, 470,939, 465,963 under Scenarios 4a, 4b, 4c, respectively (Fig. 2), decreased the peak infection (in Scenarios 3a, 3b, 3c, 4a, 4b, 4c) by at least half, and delayed the peak day by 15–75 days compared to Scenario 1 (Supplementary Table 2, Additional file 1).

**Fig. 3:**
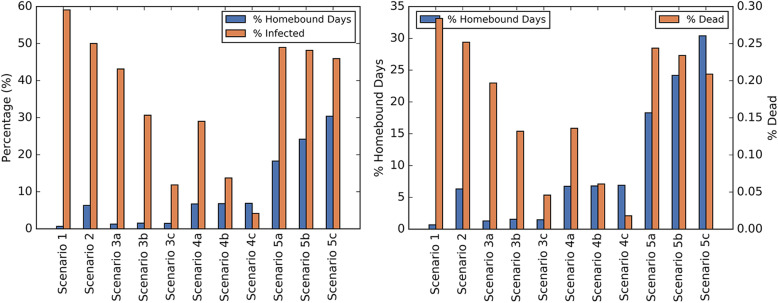
Comparison of adults homebound to infected or dead populations. Percentage of days adults homebound compared to the percentage of the population infected (left figure) and dead (right figure)

Figure 3 presents a comparison of the percentage of the population infected or dead and the percentage of days adults homebound. The percentage of the population infected was 59.09% under Scenario 1 (no intervention) and 50.02% under Scenario 2 (school closure only). The percentage of the population infected reduced to a range of 11.86–43.16% under Scenarios 3a, 3b, 3c (voluntary quarantine) and 4.15–29.02% under Scenarios 4a, 4b, 4c (voluntary quarantine with school closure). The percentage of days adults homebound was 0.68% under Scenario 1 and 6.33% under Scenario 2 (school closure only). The percentage of days adults homebound ranged from 1.30–1.55% and 6.74–6.90% under Scenarios 3a, 3b, 3c (voluntary quarantine) and Scenarios 4a, 4b, 4c (voluntary quarantine with school closure), respectively. Compared to Scenario 2 (school closure only), Scenarios 5a, 5b, 5c (shelter-in-place with school closure) reduced the percentage of the total population infected from 50.02% to 45.93–48.97% but more than doubled the percentage of days adults homebound to a range of 18.30–30.39%. Supplementary Table 3, Additional file 1 provides the percentage of days children, youth, adults, and elderly are homebound across all scenarios. Given these results, we accept Hypotheses 1 and 2. That is, voluntary quarantine (with school closure) leads to a significant reduction in the number of COVID19 infections and shelter-in-place has a minimal impact on the number of COVID19 infections while significantly increasing the number homebound.

**Fig. 4:**
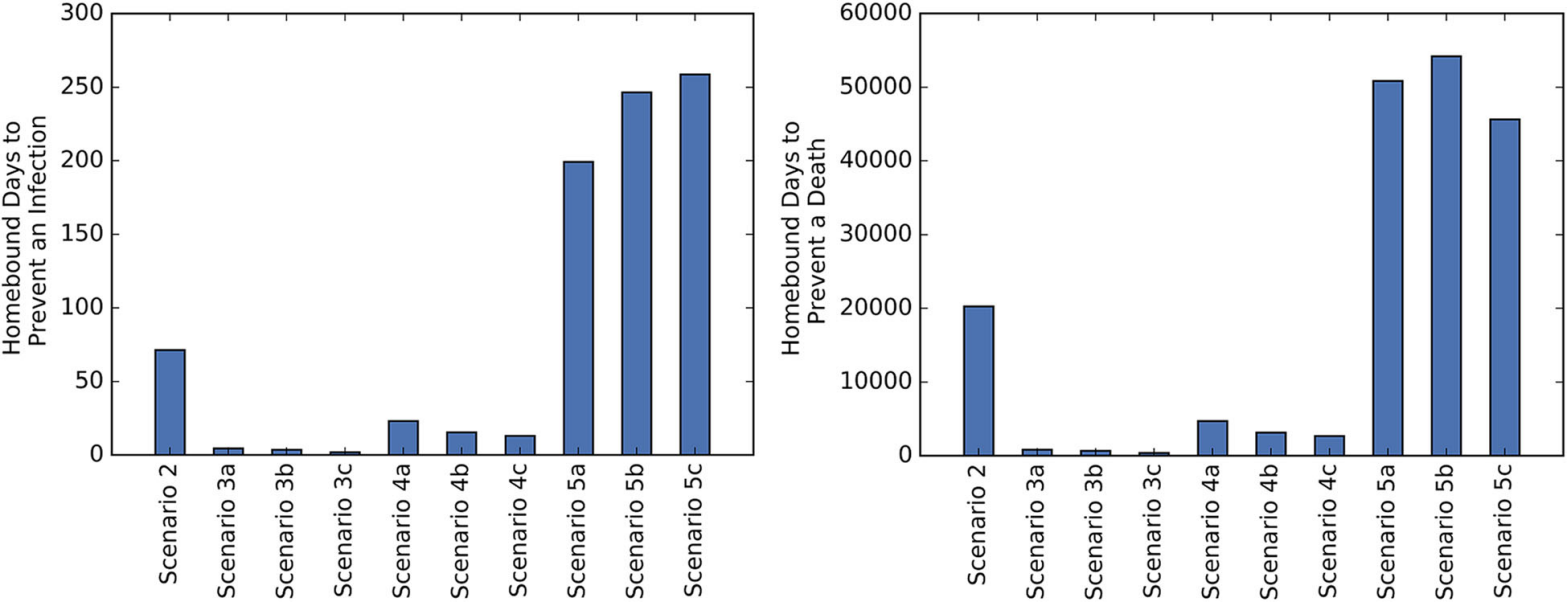
Homebound days to prevent an infection or death. Homebound days to prevent an infection (left figure) or a death (right figure)

Figure 4 presents the homebound days to prevent an infection or death. The homebound days to prevent an infection was 71 under Scenario 2 (school closure only) and over 200 under Scenarios 5a, 5b, 5c (shelter-in-place with school closure). The homebound days to prevent an infection was 1.9, 3.5, 4.5 under Scenarios 3c, 3b, 3a (voluntary quarantine), respectively, versus 13, 15.4, 23.1 under Scenarios 4c, 4b, 4a (voluntary quarantine with school closure), respectively. The homebound days to prevent a death was 20,244 under Scenario 2 (school closure only) and over 45,622 under Scenarios 5a, 5b, 5c (shelter-in-place with school closure). The homebound days to prevent a death was 383, 660, 819 under Scenarios 3c, 3b, 3a (voluntary quarantine), respectively, versus 2684, 3140, 4702 under Scenarios 4c, 4b, 4a (voluntary quarantine with school closure), respectively.

Supplementary Table 4, Additional file 1 presents the homebound and inactive peak percentages for children, youth, adults, elderly, and the total population. Increasing voluntary quarantine compliance, regardless of school closure, decreased the homebound and inactive peak percentage for adults, elderly, and the total population. Supplementary Figures 2 and 3, Additional file 1 present the homebound peak broken down by statuses for adults and elderly and for children and youth, respectively.

Supplementary Figure 4, Additional file 1 shows the percentage distribution of statuses (at home childcare, voluntary quarantine, symptoms) for the homebound peak for adults. At the homebound peak, among homebound adults: (i) Under Scenarios 2, 4a, 4b, 4c (school closure scenarios without shelter-in-place), 0.77–27.54% and 72.46–82.22% were symptomatic or providing at home childcare, respectively. (ii) Under Scenarios 3a, 3b, 3c (non-school closure scenarios), 9.61–37.85% and 0.79–3.95% were symptomatic or providing at home childcare, respectively. (iii) Under no intervention, 89.91 and 10.09% were symptomatic or providing at home childcare, respectively.

Supplementary Tables 5–7, Additional file 1 summarize the impact of voluntary quarantine, school closure and shelter-in-place by comparing the percentage difference between a pair of scenarios in terms of the homebound days (for children, youth, adult and elderly populations), cumulative infections, and deaths.

## Discussion

The COVID19 pandemic led to widespread school closure and shelter-in-place orders in the United States [27, 28]. Despite the potential public health benefits, there were many concerns about the economic impacts of shelter-in-place [10–13] and the disruptive effects of school closures on the education of children and youth [14, 21–23, 29]. This study analyzed and compared several NPI scenarios, including combinations of school closure, voluntary quarantine, and shelter-in-place, with varying compliance levels and durations, as well as baseline scenarios of no intervention (Scenario 1) and school closure only (Scenario 2).

### Main findings

Compared to no intervention, school closure reduced the percentage of the population infected by less than 16% (Supplementary Table 6, Additional file 1) while more than doubling the peak number of adults homebound and causing nearly 400,000 work absences.

Shelter-in-place combined with school closure (Scenarios 5a-5c) temporarily slowed down the infection spread and delayed the peak, but had little impact on the magnitude of the peak and the cumulative number of infections and deaths, which were similar to that observed in the school closure only scenario. However, under Scenarios 5a-5c, the peak number of homebound adults was 10–45 times larger than all other intervention scenarios.

Under voluntary quarantine (Scenarios 3a, 3b, 3c) the percentage of the population infected was 11.86–43.16% (compared to 59.09% under no intervention), with the peak number of adults homebound being 248,421-119,461. Under voluntary quarantine combined with school closure (Scenarios 4a, 4b, 4c) the percentage of the population infected was 4.15–29.02% (compared to 50.02% under school closure only), with the peak number of adults homebound being 499,188-465,963. Compared to voluntary quarantine (Scenarios 3a, 3b, 3c), voluntary quarantine combined with school closure (Scenarios 4a, 4b, 4c) reduced the percentage of population infected by at least 32% while almost doubling the peak number of adults homebound.

Compared to school closure only, voluntary quarantine combined with school closure yielded up to a 92% decrease in cumulative infections and deaths while homebound days increased by at most 9% for adults, 7% for elderly and 1.5% for the total population*.* Under voluntary quarantine scenarios, the number of homebound days to prevent an infection or death was 3–82 times lower than that of all other scenarios.

### Comparison with other studies

While recent research has assessed the impact of NPIs on reducing the spread of COVID19, there are limited studies that examine which interventions have a higher overall impact on the homebound population versus their ability to curb infection spread and adverse outcomes [1–3, 8, 9, 24, 25]. Our study aims to bridge this gap by evaluating the trade-off between the health benefits of NPIs and their potential economic and social consequences due to homebound populations.

### Implication and explanation of findings

This study found that school closure alone had limited impact on reducing the spread of COVID19. The majority of adults homebound under school closure alone were due to the need to provide at home childcare. The positive public health impact of shelter-in-place came at a very high societal cost. In contrast, high levels of voluntary quarantine compliance decreased the percentage of the population infected and the peak number of adults homebound (or absent from work). Voluntary quarantine compliance provided the greatest benefits in terms of the reduction in infections and deaths compared to the number of adults homebound.

### Strengths and limitations

Some of the conclusions of this study may be generalized to other states/countries that have geographic and population characteristics similar to the state of Georgia. The model and analysis would need to be adjusted for other pandemics; for example, COVID19 leads to fewer adverse health outcomes in younger populations and this may explain why school closure has a lesser impact on reducing infection spread. If facemask usage was also considered in the NPI scenarios, the relative reduction in the number of cases and deaths could be higher compared to baseline scenarios. The simulation was populated with data from the state of Georgia and the results presented may not apply to other states or regions which have significantly different population characteristics or density.

## Conclusion

Many governments are faced with difficult decisions about when and how quickly to lift social distancing restrictions and reopen their economies; hence, it is crucial to analyze the benefits of NPIs in decreasing the spread of COVID19 versus the economic and social consequences considering the people who become homebound due to illness or due to complying with NPIs.

It is important to take into account the measures considered in this study when making decisions based on NPIs, in light of the fact that most decisions are made based on the number of infections alone.

While large-scale interventions such as shelter-in-place temporarily slow down the infection spread, they are highly disruptive to the society and their public health impact is limited unless they are imposed for long durations of time, with high compliance levels, or followed by additional interventions.

Targeted interventions such as voluntary quarantine or voluntary quarantine combined with school closure significantly reduce the infection spread without causing a social and economic disruption as in the case of an extended shelter-in-place.

### Recommendations

Strong public messaging should continue about voluntary quarantine, voluntary shelter-in-place (if possible), as well as other practices of physical distancing and the usage of facemasks.

### Future directions

Further research could examine the effect of facemask usage on the impact of NPIs, as well as continue to explore various combinations of NPI strategies. As we approach the release of a potential COVID19 vaccine, another important research question to consider is the impact of vaccine availability and allocation strategies on the infection spread and homebound populations, along with other NPIs.

## Supplementary Information

**Additional file 1.** Modeling peer-to-peer interactions and supplemental figures, tables, and results; provides details on how health status (e.g., symptomatic, hospitalized, dead) and homebound status of each household member is tracked in the simulation along with supplementary tables, figures, and results that aid in the discussion in the main text.

## Data Availability

The datasets generated and then analyzed during the current study are available from the corresponding author on reasonable request. All data was publicly available and can be accessed through the U.S. Census Bureau [30–32], U.S. Department of Labor [33], and the New York Times [34].
