# Supplementary material for: Homebound by COVID19: the benefits and consequences of non-pharmaceutical intervention strategies
Source: BMC Public Health. 2021 Apr 6;21:655. doi: 10.1186/s12889-021-10725-9 (PMC8022402; doi:10.1186/s12889-021-10725-9)
Supplement: Supplementary file 1 — Additional file 1. Modeling peer-to-peer interactions and supplemental figures, tables, and results; provides details on how health status (e.g., symptomatic, hospitalized, dead) and homebound status of each household member is tracked in the simulation along with supplementary tables, figures, and results that aid in the discussion in the main text. [file 12889_2021_10725_MOESM1_ESM.docx]

**Homebound by COVID19: The Benefits and Consequences of Non-Pharmaceutical Intervention Strategies Supplementary Material**

**Supplementary Section A: Modeling Peer-to-Peer Interactions**

In the state of Georgia there are 1,336,490 children, 1,418,910 youths, 6,685,870 adults, and 1,356,730 elderly [1]. The agent-based simulation model was populated with the population-related data, including demographic information, travel patterns, and household statistics, from the state of Georgia [2]. The population in the simulation is reflective of the population characteristics of Georgia. For computational efficiency, we assumed an agent consists of 10 people of the same age, hence the simulated population is about 1/10^th^ of the population in the state, following the same age distribution.

Children (ages 0-9) and youth (ages 10-19) were considered homebound under any combination of the following settings: symptomatic, complying with voluntary quarantine, or school closure. *Compliance with voluntary quarantine* is defined as all household members staying home if there is a person with cold/flu like symptoms in the household, until the entire household is symptom-free. Adults (ages 20-64) and elderly (ages 65+) were considered homebound under any combination of the following settings: at home childcare, symptomatic, complying with voluntary quarantine, or shelter-in-place. *At home childcare* is defined as providing supervision to a child who is home due to their status. *Supplementary Table 1* provides a list of statuses considered for each age group.

**Supplementary Table 1. Population statuses for tracking.** Main statuses for tracking children, youth, adults and elderly populations.

|  |  | **Homebound** | | | | |  |  |  |
| --- | --- | --- | --- | --- | --- | --- | --- | --- | --- |
|  | **Active** | **SC** | **Symptoms** | **VQ** | **SIP** | **At Home**  **Childcare** | **Hospital Care** | **Hospitalized** | **Dead** |
| **Children** | ✓ | ✓ | ✓ | ✓ |  |  |  | ✓ | ✓ |
| **Youth** | ✓ | ✓ | ✓ | ✓ |  | ~ |  | ✓ | ✓ |
| **Adult** | ✓ |  | ✓ | ✓ | ✓ | ✓ | ✓ | ✓ | ✓ |
| **Elderly** | ✓ |  | ✓ | ✓ | ✓ | ✓ | ✓ | ✓ | ✓ |

In the simulation scenarios, this study accounted for the following recommendations from the Georgia Division of Family and Children Services [3]: children ages 0-8 require at-home supervision or childcare, children ages 9-12 require partial at-home supervision, and children ages 13-19 do not require at-home supervision and can provide childcare to the younger child population.

A child needed supervision if school was closed, they were complying with voluntary quarantine, or they were symptomatic. Note that several of these situations could occur at the same time, e.g., a child was symptomatic and school was closed. Since supervision was the only care considered, a child was assumed to be supervised if a youth, adult, or elderly family member already had homebound status for other reasons (e.g., symptomatic, voluntary quarantine, shelter-in-place). That is, if at least one caregiver of the appropriate age (youth, adult, or elderly family member) was homebound in any of the capacities listed above, then no more supervision was needed at home. In the remainder of the supplementary material, reference to “at home childcare” or “care” means that the child received at home supervision. Based on availability, it was assumed that youth were the first to provide care, then elderly, and lastly adults, considering adult participation in the workforce. However, if an adult or elderly was already homebound for other reasons and care was needed, the adult was designated as providing care, then elderly, and lastly youth.

Similarly, for any hospitalized minor (person between the ages of 0 and 19), it was assumed that an adult or elderly family member would provide supervision (referred to as hospital care).

**Supplementary Section B: Supplemental Figures, Tables, and Results**

Below are supplementary tables, figures, and results that aid in the discussion in the main text. Definitions for homebound and inactive statuses are provided in the Modeling Case Projections and Estimating Intervention Impact section of the main text.

The following terminology and abbreviations are used in the remainder of this document:

- *SC*: school closure;
- *VQ*: voluntary quarantine;
- *SIP*: shelter-in-place;
- *Symptoms*: symptomatic infection; and
- *Symptoms & VQ*: symptomatic infection and complying with voluntary quarantine.

**Supplementary Table 2. Outcome measures.** Statistical summary that compares baseline and intervention scenarios with respect to cumulative deaths, cumulative infections, percentage of the population infected (infection attack rate, IAR %), peak infection (%), and peak day.

|  | **Cumulative Deaths** | **Cumulative**  **Infections** | **IAR**  **(%)** | **Peak**  **Infection (%)** | **Peak**  **Day** |
| --- | --- | --- | --- | --- | --- |
| **Scenario 1** | 30638 | 6381089 | 59.09 | 1.657 | 62 |
| **Scenario 2** | 27186 | 5400991 | 50.02 | 1.059 | 77 |
| **Scenario 3a** | 21291 | 4660731 | 43.16 | 0.795 | 77 |
| **Scenario 3b** | 14268 | 3310478 | 30.66 | 0.403 | 93 |
| **Scenario 3c** | 4964 | 1280719 | 11.86 | 0.102 | 122 |
| **Scenario 4a** | 14705 | 3133277 | 29.02 | 0.341 | 110 |
| **Scenario 4b** | 6568 | 1483928 | 13.74 | 0.120 | 126 |
| **Scenario 4c** | 1954 | 447647 | 4.15 | 0.029 | 137 |
| **Scenario 5a** | 26354 | 5287339 | 48.97 | 0.943 | 111 |
| **Scenario 5b** | 25269 | 5201577 | 48.17 | 0.908 | 129 |
| **Scenario 5c** | 22583 | 4959530 | 45.93 | 0.875 | 148 |

It is estimated that 71% of adults would not be able to work from home [4]. Across all scenarios, *Supplementary Figure 1* presents the number of adults who are absent from work over time, which was estimated by aggregating the number of adults who were hospitalized or provided hospital care, plus 71% of homebound adults. Under Scenarios 1, 3a, 3b, 3c (non-school closure scenarios), the peak number of adults absent from work decreased from 188,959 under Scenario 3a to 86,472 under Scenario 3c, and the peak under Scenario 1 was 185,338. Under Scenarios 2, 4a, 4b, 4c, 5a, 5b, 5c (school closure scenarios), the peak number of adults absent from work was highest under Scenarios 5a, 5b, 5c, due to shelter-in-place, ranging from 3,827,043 to 3,826,248 followed by a peak of 385,642 under Scenario 2. Higher compliance with voluntary quarantine reduced peak adults absent from work to 360,774, 336,580, and 331,358 in Scenarios 4a, 4b, 4c, respectively.

**Supplementary Figure 1. Adults absent from work over time.** Number of adults who are absent from work over time.


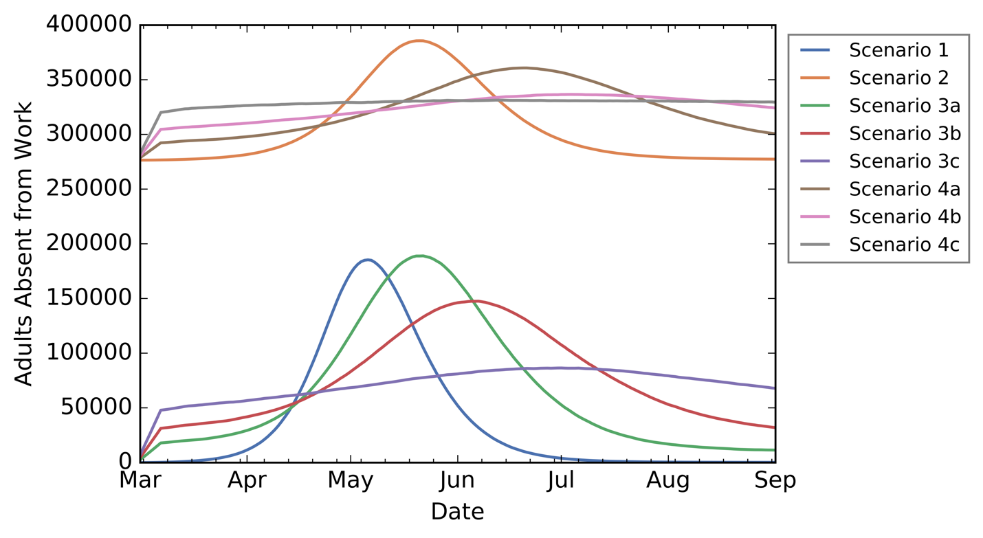


**Supplementary Table 3. Percentage of days homebound.** Percentage of days children, youth, adults, and elderly homebound.

| Homebound Days | | | | |
| --- | --- | --- | --- | --- |
|  | **Children %** | **Youth %** | **Adults %** | **Elderly %** |
| **Scenario 1** | .63 | .70 | .68 | .58 |
| **Scenario 2** | 100 | 100 | 6.33 | 11.57 |
| **Scenario 3a** | 1.52 | 1.57 | 1.30 | 1.23 |
| **Scenario 3b** | 2.04 | 2.08 | 1.55 | 1.51 |
| **Scenario 3c** | 2.04 | 2.08 | 1.48 | 1.48 |
| **Scenario 4a** | 100 | 100 | 6.74 | 12.04 |
| **Scenario 4b** | 100 | 100 | 6.79 | 12.18 |
| **Scenario 4c** | 100 | 100 | 6.90 | 12.37 |
| **Scenario 5a** | 100 | 100 | 18.30 | 22.70 |
| **Scenario 5b** | 100 | 100 | 2419 | 28.17 |
| **Scenario 5c** | 100 | 100 | 30.39 | 33.95 |

**Supplementary Table 4. Homebound and inactive peak percentages.** Homebound and inactive peak percentage for children, youth, adults, elderly, and the total population.

| Inactive | | | | | |
| --- | --- | --- | --- | --- | --- |
|  | **Children %** | **Youth %** | **Adult %** | **Elderly %** | **Total %** |
| **Scenario 1** | 3.19 | 3.58 | 3.75 | 3.27 | 3.58 |
| **Scenario 2** | 100.00 | 100.00 | 8.01 | 12.65 | 32.06 |
| **Scenario 3a** | 4.52 | 4.74 | 3.90 | 3.56 | 4.04 |
| **Scenario 3b** | 4.04 | 4.18 | 3.07 | 2.86 | 3.30 |
| **Scenario 3c** | 2.53 | 2.60 | 1.81 | 1.78 | 2.00 |
| **Scenario 4a** | 100.00 | 100.00 | 7.56 | 12.64 | 31.78 |
| **Scenario 4b** | 100.00 | 100.00 | 7.08 | 12.40 | 31.46 |
| **Scenario 4c** | 100.00 | 100.00 | 6.98 | 12.44 | 31.40 |
| **Scenario 5a** | 100.00 | 100.00 | 80.60 | 80.77 | 85.57 |
| **Scenario 5b** | 100.00 | 100.00 | 80.61 | 80.76 | 85.58 |
| **Scenario 5c** | 100.00 | 100.00 | 80.62 | 80.80 | 85.59 |
| Homebound | | | | | |
|  | **Children %** | **Youth %** | **Adult %** | **Elderly %** | **Total %** |
| **Scenario 1** | 3.19 | 3.58 | 3.37 | 2.82 | 3.30 |
| **Scenario 2** | 100.00 | 100.00 | 7.73 | 12.33 | 31.85 |
| **Scenario 3a** | 4.52 | 4.73 | 3.72 | 3.33 | 3.89 |
| **Scenario 3b** | 4.03 | 4.17 | 2.97 | 2.74 | 3.23 |
| **Scenario 3c** | 2.53 | 2.60 | 1.79 | 1.75 | 1.98 |
| **Scenario 4a** | 100.00 | 100.00 | 7.47 | 12.53 | 31.71 |
| **Scenario 4b** | 100.00 | 100.00 | 7.04 | 12.36 | 31.43 |
| **Scenario 4c** | 100.00 | 100.00 | 6.97 | 12.43 | 31.39 |
| **Scenario 5a** | 100.00 | 100.00 | 80.59 | 80.76 | 85.56 |
| **Scenario 5b** | 100.00 | 100.00 | 80.60 | 80.75 | 85.57 |
| **Scenario 5c** | 100.00 | 100.00 | 80.61 | 80.79 | 85.58 |

**Supplementary Figure 2. Homebound peaks for adults and elderly broken down by statuses.** Homebound peak for adults (left), and elderly (right) broken down by statuses.


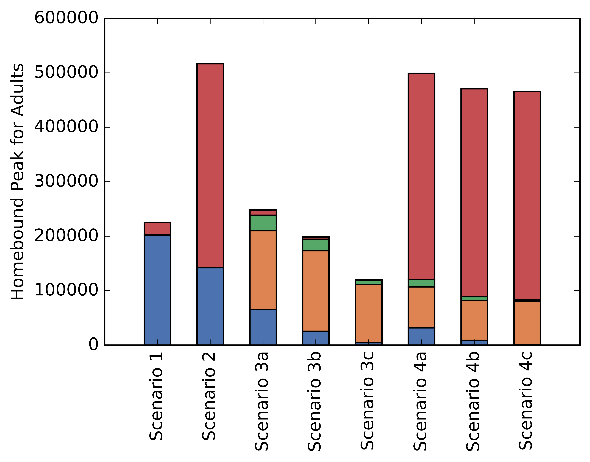

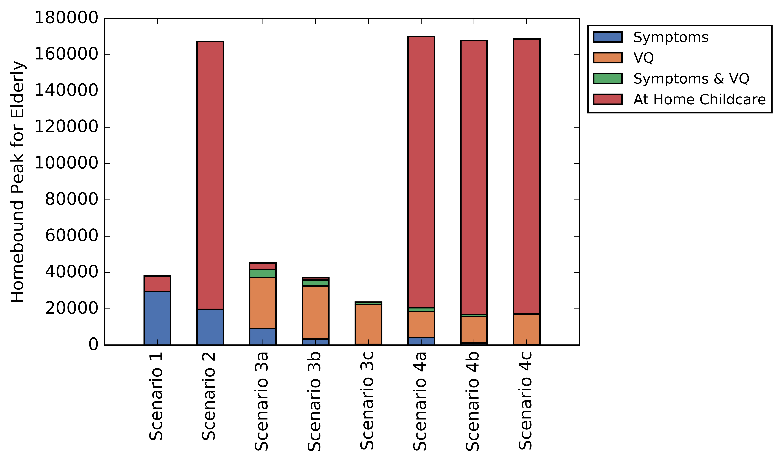


**Supplementary Figure 3. Homebound peaks for children and youth broken down by statuses.** Homebound peak for children (left), and youth (right) broken down by statuses.


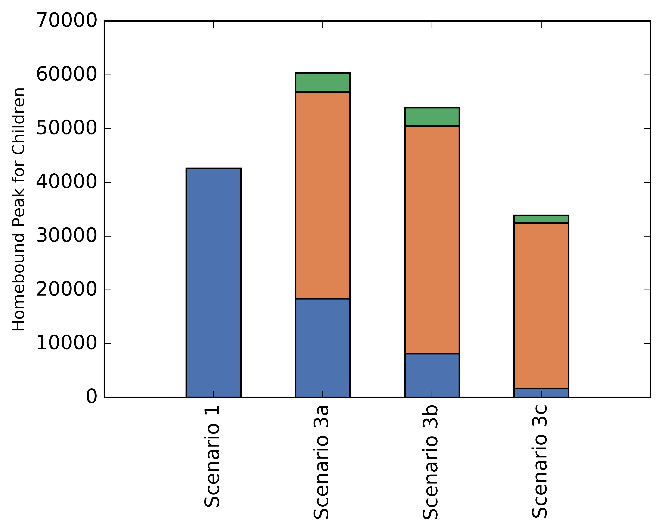

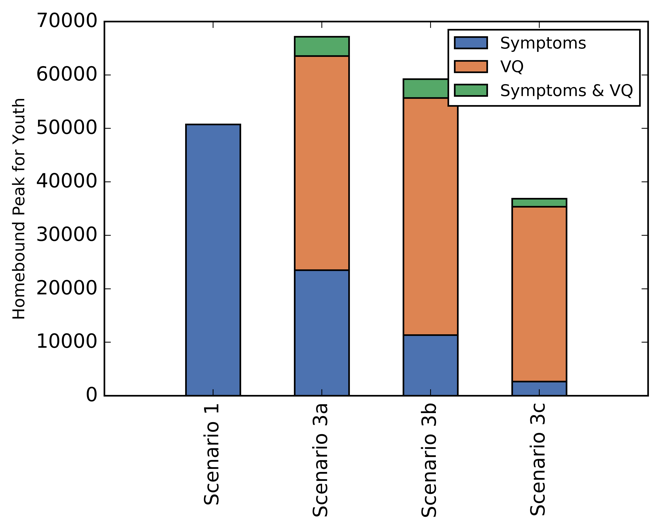


**Supplementary Figure 4. Distribution of statuses for homebound peak for adults.** Percentage distribution of statuses for homebound peak for adults.


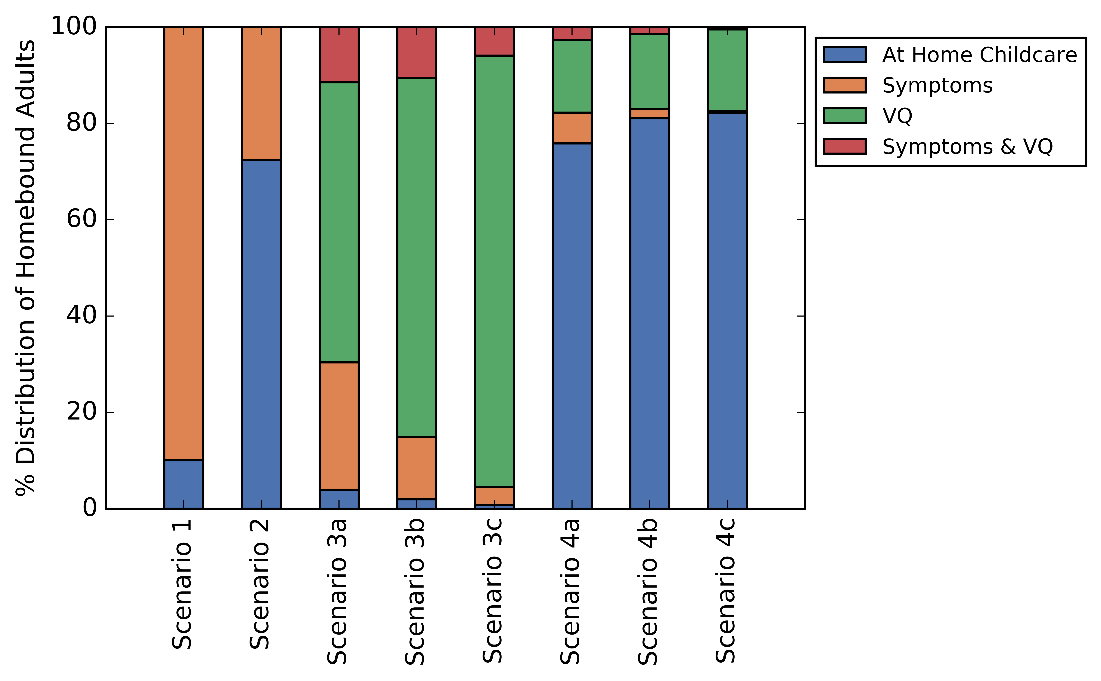


Under school closure, having voluntary quarantine or increasing voluntary quarantine compliance yielded up to a 93% decrease in cumulative infections and deaths while homebound days increased by at most 9% for adults, 7% for elderly, and 1.5% for the total population (*Supplementary Table 5*).

Under non-school closure, having voluntary quarantine or increasing voluntary quarantine compliance yielded up to an 80% and 84% decrease in cumulative infections and deaths, respectively, while homebound days increased at most by 129% for adults, 161% for elderly, and 153% for the total population (*Supplementary Table 5*).

**Supplementary Table 5. Effect of increasing voluntary quarantine compliance.** Change in the cumulative infections and deaths compared to the change in homebound days with respect to increase in voluntary quarantine compliance, from 0% to 50%, 70% and 90% (Scenario 1 vs Scenarios 3a, 3b, 3c and Scenario 2 vs Scenarios 4a, 4b, 4c), 50% to 70% and 90% (Scenario 3a vs Scenarios 3b, 3c and Scenario 4a vs Scenarios 4b, 4c), 70% to 90% (Scenario 3b vs Scenario 3c and Scenario 4b vs Scenario 4c).

|  | **% Change in Homebound Days** | | | | **% Change in**  **Cumulative**  **Infections** | **% Change in**  **Cumulative**  **Deaths** |
| --- | --- | --- | --- | --- | --- | --- |
|  | **Children and Youth** | **Adult** | **Elderly** | **Total Pop.** |  |  |
| **Scenario 1 vs 3a** | 133.00 | 91.10 | 113.77 | 104.28 | -26.96 | -30.51 |
| **Scenario 1 vs 3b** | 210.90 | 128.54 | 160.98 | 153.11 | -48.12 | -53.43 |
| **Scenario 1 vs 3c** | 211.61 | 117.12 | 156.26 | 145.52 | -79.93 | -83.80 |
| **Scenario 3a vs 3b** | 33.43 | 19.59 | 22.08 | 23.90 | -28.97 | -32.98 |
| **Scenario 3a vs 3c** | 33.74 | 13.61 | 19.87 | 20.19 | -72.52 | -76.68 |
| **Scenario 3b vs 3c** | 0.23 | -5.00 | -1.81 | -3.00 | -61.31 | -65.21 |
| **Scenario 2 vs 4a** | 0.00 | 6.44 | 4.13 | 1.01 | -41.99 | -45.91 |
| **Scenario 2 vs 4b** | 0.00 | 7.27 | 5.35 | 1.18 | -72.52 | -75.84 |
| **Scenario 2 vs 4c** | 0.00 | 9.07 | 6.98 | 1.48 | -91.71 | -92.81 |
| **Scenario 4a vs 4b** | 0.00 | 0.78 | 1.18 | 0.16 | -52.64 | -55.34 |
| **Scenario 4a vs 4c** | 0.00 | 2.47 | 2.73 | 0.47 | -85.71 | -86.71 |
| **Scenario 4b vs 4c** | 0.00 | 1.68 | 1.54 | 0.30 | -69.83 | -70.25 |

From voluntary quarantine without school closure (Scenarios 3a, 3b, 3c) to voluntary quarantine with school closure (Scenarios 4a, 4b, 4c), school closure had the least impact to homebound days in medium compliance (comparing Scenario 3b to 4b); however, the greatest reduction in cumulative infections and deaths was obtained in high compliance (comparing Scenario 3c to 4c). With school closure, homebound days could increase 3-9 and 7-20 times for adults and elderly, respectively. Due to voluntary quarantine, school closure was less disruptive when comparing the change in homebound days from Scenarios 3a, 3b, 3c to 4a, 4b, 4c with the change in homebound days from Scenario 1 to Scenario 2. School closure had the worst impact on homebound days but could provide up to a 61% decrease in cumulative infections and deaths. The impact of school closure on cumulative infections and deaths increased with increasing voluntary quarantine compliance (*Supplementary Table 6*).

**Supplementary Table 6. Effect of school closure.** Change in the cumulative infections and deaths versus the change in homebound days with respect to school closure.

|  | **% Change in Homebound Days** | | | | **% Change in**  **Cumulative Infections** | **% Change in**  **Cumulative Deaths** |
| --- | --- | --- | --- | --- | --- | --- |
|  | **Children and Youth** | **Adult** | **Elderly** | **Total Pop.** |  |  |
| **Scenario 1 vs 2** | 14999.13 | 831.49 | 1903.09 | 4564.12 | -15.36 | -11.27 |
| **Scenario 3a vs 4a** | 6380.44 | 418.81 | 875.71 | 2206.36 | -32.77 | -30.93 |
| **Scenario 3b vs 4b** | 4756.89 | 337.21 | 708.63 | 1764.45 | -55.17 | -53.97 |
| **Scenario 3c vs 4c** | 4745.83 | 367.95 | 736.20 | 1827.91 | -65.05 | -60.64 |

Compared to voluntary quarantine, shelter-in-place combined with school closure impacted a larger population by increasing homebound days greatly (*Supplementary Tables 5 and 7*, Scenario 2 vs 4a, 4b, 4c and Scenario 2 vs 5a, 5b, 5c). However, shelter-in-place with school closure scenarios did not lead to a considerable decrease in cumulative infections and deaths compared to voluntary quarantine with school closure scenarios (Scenario 4a, 4b, 4c). Compared to school closure only (Scenario 2), shelter-in-place combined with school closure lead to at most a 8.2% and 17% decrease in cumulative infections and deaths, respectively, while increasing homebound days for adults by at least 1.9 folds. On the other hand, compared to Scenario 2, even low voluntary quarantine compliance with school closure (Scenario 4a) could provide at least a 42% decrease in cumulative infections and deaths while increasing homebound days for adults by just 6.44%.

**Supplementary Table 7. Effect of shelter-in-place durations.** Change in the cumulative infections and deaths versus the change in homebound days with respect to varying durations of shelter-in-place.

|  | **% Change in Homebound Days** | | | **% Change in**  **Cumulative**  **Infections** | **% Change in**  **Cumulative**  **Deaths** |
| --- | --- | --- | --- | --- | --- |
|  | **Adult** | **Elderly** | **Total Pop.** |  |  |
| **Scenario 2 vs 5a** | 189.06 | 96.25 | 28.52 | -2.10 | -3.06 |
| **Scenario 2 vs 5b** | 282.22 | 143.53 | 42.56 | -3.69 | -7.05 |
| **Scenario 2 vs 5c** | 380.14 | 193.53 | 57.34 | -8.17 | -16.93 |
| **Scenario 5a vs 5b** | 32.23 | 24.09 | 10.93 | -1.62 | -4.12 |
| **Scenario 5a vs 5c** | 66.10 | 49.57 | 22.43 | -6.20 | -14.31 |
| **Scenario 5b vs 5c** | 25.62 | 20.53 | 10.37 | -4.65 | -10.63 |

**REFERENCES**

1. U.S. Census Bureau: **American Community Survey, 2018 American Community Survey 1-year Estimates**. In*.*: U.S. Census Bureau; 2018.

2. Keskinocak P, Oruc BE, Baxter A, Asplund J, Serban N: **The impact of social distancing on COVID19 spread: State of Georgia case study**. *PLoS One* 2020, **15**(10):e0239798.

3. **Child Supervision Guidelines in Georgia** [<https://abuse.publichealth.gsu.edu/child-supervision-guidelines-in-georgia/>]

4. U.S. Department of Labor: **Job Flexibilities and Work Schedules -- 2017-2018 Data From The American Time Use Survey**. In*.*; 2019.
